# Assessment of Indoor Air Quality of Four Primary Health Care Centers in Qatar

**DOI:** 10.3390/microorganisms10102055

**Published:** 2022-10-18

**Authors:** Hana Abdelrahman, Lubna Abu-Rub, Hassan Al Mana, Yousef Alhorr, Asmaa Al Thani, Hamda Qotba, Hadi M. Yassine, Nahla O. Eltai

**Affiliations:** 1 Biomedical Research Center, Qatar University, Doha P.O. Box 2713, Qatar; 2 Gord, Gulf Organization for Research and Development, Doha P.O. Box 210162, Qatar; 3 Research Center, Clinical affairs, Primary Health Care Center, Doha P.O. Box 26555, Qatar

**Keywords:** airborne bacteria, indoor environment, PHCC, total bacterial count, seasonal variation, indoor air quality

## Abstract

Airborne bacteria pose a potential risk to human health upon inhalation in the indoor environments of health care facilities. Airborne bacteria may originate from various sources, including patients, workers, and daily visitors. Hence, this study investigates the quantity, size, and identification of airborne bacteria indoors and outdoors of four Primary Health Care Centers (PHCC) in Doha, Qatar. Air samples were collected from the lobby, triage room, and outside environment of the centers, including, Qatar University (QU-HC), Al-Rayyan (AR-HC), Umm-Ghuwailina (UG-HC), and Old Airport (OA-HC) between August 2020 and March 2021, throughout both the hot and the cold seasons. Samples were collected using an Anderson six-stage cascade impactor. The mean of the total colony-forming units was calculated per cubic meter of air (CFU/m^3^). QU-HC had the lowest mean of total bacterial count compared with other centers in the indoor and outdoor areas with 100.4 and 99.6 CFU/m^3^, respectively. In contrast, AR-HC had the highest level, with 459 CFU/m^3^ indoors, while OA-HC recorded the highest bacterial concentration of the outdoor areas with a total mean 377 CFU/m^3^. In addition, 16S rRNA sequencing was performed for genera identification. *Staphylococcus*, *Acinetobacter*, *Bacillus*, and *Pseudomonas* were the four most frequently identified bacterial genera in this study. The abundance of airborne bacteria in the four health centers was higher in the cold season. About 46% of the total airborne bacterial count for three PHCC centers exceeded 300 CFU/m^3^, making them uncompliant with the World Health Organization’s (WHO) recommendation for indoor settings. Consequently, an IAQ standards should be shaped to establish a baseline for measuring air pollution in Qatar. Additionally, it is crucial to understand seasonal fluctuations better so that hospitals can avoid rising and spreading infection peaks.

## 1. Introduction

Concerns about airborne infections have increased significantly after the COVID-19 pandemic. Airborne microbes are some of the most important indoor air quality (IAQ) complications for public and occupational health [1]. Health care facilities are considered critical environments and a regular assessment of IAQ is essential to create safe environments and to ensure public health [2]. In health care facilities, contamination usually spreads through air droplets or by direct touch [3]. The magnitude of this spread is affected by several environmental factors, including the number of occupants (patients, visitors, and workers), building features, humidity levels, and ventilation systems [4]. Air contaminants may contain pathogens that can cause infections in hospitalized patients. The severity of these airborne infections depends on the bioaerosol concentration, exposure time, and the individual immune system strength [5]. This is of concern mainly to patients with compromised immune systems, such as those in intensive care units or oncology wards [6,7,8]. Accordingly, it is necessary to identify airborne microbe species and their prevalence in various health settings to guide infection prevention and control measures and to mitigate transmission risk.

Numerous papers have been published about airborne microbes in hospital environments [2,9,10,11]. Studies on bacterial concentrations in the air show a considerable variation in abundance and species diversity between locations. Nevertheless, some species are commonly found in most sites, including *Staphylococcus*, *Bacillus*, and *Micrococcus* [9,11,12,13]. Airborne transmission is a known route for multiple highly threatening infectious microbes, such as methicillin-resistant *Staphylococcus aureus* (MRSA), *Mycobacterium tuberculosis*, and multi-drug-resistant *Acinetobacter* spp., that have been isolated from health care facility environments [14,15,16].

The World Health Organization (WHO) recommends that the total bacterial loads not exceed 1000 CFU/m^3^ in indoor environments and work environments should not exceed 300 CFU/m^3^ [17]. However, in the hospital setting, the total bacterial concentration should be less than 100 CFU/m^3^ in an area occupied by immunocompromised people [12,18]. A recent systematic review found frequent non-compliance with the WHO recommendation and highlighted the urgent need to define robust strategies and international standards to enhance IAQ in health care facilities [19,20]. Legislation governing IAQ standards has not been developed or implemented in Qatar [20]. Thus, this study aims to assess the levels of airborne bacteria of different Primary Health Care Centers (PHCCs) in the state of Qatar and to compare the concentrations and composition between the hot and cold seasons to provide a baseline upon which an IAQ framework can be built. In addition to the total bacterial concentration and diversity, the study assessed the prevalence of MRSA in the PHCC air samples, since it is commonly found in hospitals and leads to a severe invasive infection.

## 2. Materials and Methods

### 2.1. Sampling Period and Sites

**Figure 1:**
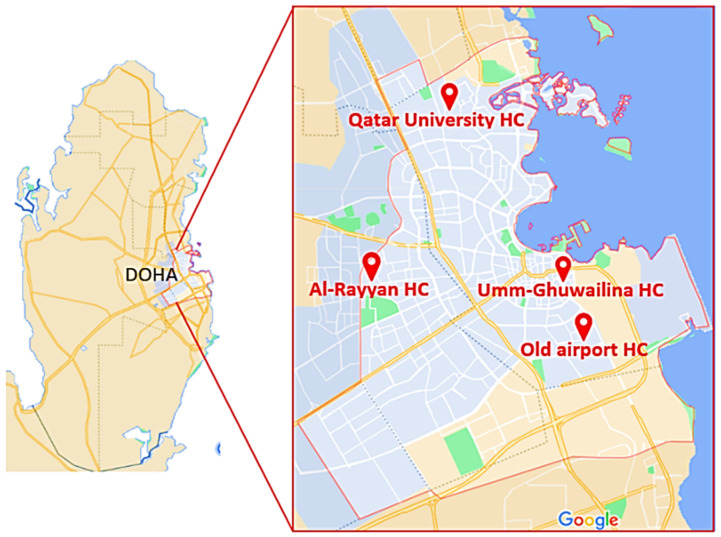
Map of Qatar showing the locations of the four PHCCs in Doha city where samples were collected: Qatar University, Al-Rayyan, Umm-Ghuwailina, and Old Airport health centers.

**Figure 2:**
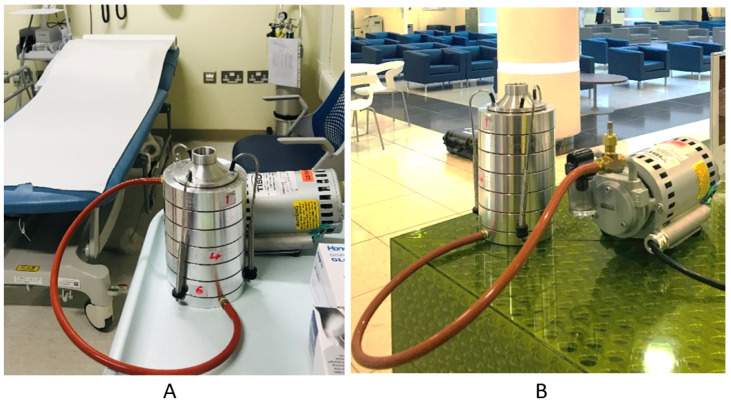
Image representing sampling location of the indoor area at QU-HC using the Andersen impactor. (**A**) Triage room. (**B**) Lobby.

Samples were collected between August 2021 and March 2021. The samples were collected from four PHCCs in the state of Qatar located in Doha city: Qatar University Health Center (QU-HC) (25°22′27.0″ N 51°28′47.5″ E), located in Al Tarfa district; Umm-Ghuwailina Health Center (UG-HC) (25°16′36.9″ N 51°32′42.8″ E), located at Umm Ghuwailina district; Old Airport Health Center (OA-HC) (25°15′24.3” N 51°33′27.0″ E), located at Old Airport district; Al-Rayyan Health Center (AR-HC) (25°16′47.8″ N 51°25′55.8″ E), located at Al-Rayyan municipality (Figure 1). For each PHCC, samples were collected twice per month, except February and March 2021 when the sites were only visited once because of COVID pandemic restrictions. Collection of the samples was carried out in the mornings between 8 AM and 11 AM from three sublocations, the lobby, the triage room (indoor area), (Figure 2) and the outdoors. All PHCCs were established in the early 1990s, except for QU-HC which was recently completed and opened for patients in 2018. It is located in an area of 19,000 square meters, with a capacity to accommodate 35,000 patients [21]. The area size of the sublocation triage room at QU-HC was 36 m^2^ and the lobby was 150 m^2^, whereas the triage room and the lobby areas in UG-HC, OA-HC, and AR-HC were approximately 46 m^2^ and 16 m^2^, respectively, with a capacity to accommodate approximately 10,000 patients [21]. All PHCCs were ventilated by a central ventilation system undergoing biannual filter replacement and maintenance. None of the PHCCs had natural ventilation.

### 2.2. Cultivable Airborne Bacteria

#### 2.2.1. Sampling Instrument

Researchers from Qatar University Biomedical Research Center collected samples with an Andersen six-stage cascade impactor (TE-10-800, Tisch Environmental, Italy) (Figure 2), employing an active size-resolved cultivable airborne bacterium. The Anderson impactor isolates airborne bacteria according to their aerodynamic diameter and gathers microorganisms on an agar plate. Size resolution occurs through six stages of reducing pore size: stage 1:> 7.0 μm, stage 2: 7.0–4.7 μm, stage 3: 4.7–3.3 μm, stage 4: 3.3–2.1 μm, stage5: 2.1–1.1 μm, and stage 6: 1.1–0.65 μm. The stages reflect the deposition location of inhalable bacteria in the human lungs. The sum of stages 1 and 2 represents the fraction potentially depositing in the upper airways, and stages 3–6 denote respirable bacteria [22].

In each instance, duplicate samples were collected using two Andersen impactor devises at a time, each loaded with six nutrient agar plates (90 mm diameter size) (HiMedia, India). The devices were positioned 1.5 m above the floor and 1 m away from the wall to represent the human breathing zone. The average temperature of the indoor and outdoor areas ranged between (20–25 °C) and (27–45 °C), respectively. For the outdoors, the humidity (56.5%–13%) was frequently measured along with the sample collection. Before and between each sampling location, the inside of the sampler and the cap of the cascade were cleaned with a 70% ethanol solution to prevent cross-contamination. The samples were collected at a flow rate of 28.3 L/min for 15 min. All plates were immediately transferred into the lab and incubated at 37 °C for three days. Then, the colonies on each plate were counted for each stage by the researchers and then collected in 1.5 mL tubes containing 20% glycerol and stored at −20 °C to be identified by 16S rRNA sequencing. The concentrations of microorganisms (CFU/m^3^) per stage were calculated as follows [23].

$$\mathrm{CFU}/m^{3}=\frac{\mathrm{average}\;\mathrm{number}\;\mathrm{of}\;\mathrm{CFU}\;\mathrm{at}\;\mathrm{the}\;\mathrm{stage}\;*1000}{\mathrm{sampling}\;\mathrm{flow}\;\mathrm{rate}\;\left(\frac{L}{\min}\right)\;*\;\mathrm{sampling}\;\mathrm{time}}$$

#### 2.2.2. Pooling

The cultivable air bacteria samples were pooled for more feasible sequencing by combining all six stages from the Anderson impactor per indoor sublocation per season (hot and cold), as shown in Table 1, since the state of Qatar has mainly two seasons: the hot season lasts from March to September, and the cold season lasts from October to February.

#### 2.2.3. DNA Extraction and 16S rRNA Sequencing

The genomic DNA was extracted from the pooled cultivable samples using QIAamp^®^ UCP pathogen minikit (Qiagen, Germany) following the manufacturer’s protocol and then quantified using the NanoDrop device (NanoDrop lite spectrometer, Thermo Fisher, US). The genomic DNA was sent to the Beijing Genomics Institute (BGI; Hong Kong, China) for 16S rRNA sequencing. Briefly, libraries were constructed by amplifying the16S rRNA with polymerase chain reaction (PCR), purifying the amplicons using Agencourt AMPure XP beads, and labeling them. The library sizes and concentrations were detected with an Agilent 2100 bioanalyzer; sequencing was performed on the HiSeq platform (Illumina) according to the insert size. The resulting raw reads were filtered to generate high-quality clean reads using the iTools Fqtools fqcheck software (v.0.25).

#### 2.2.4. Statistical Analysis and 16S rRNA Bioinformatics

Data were entered into Microsoft Excel 2010 (Microsoft Corporation, New York, NY, USA) for data collection. SPSS statistics Version 26 (Statistical Package for the Social Science; SPSS Inc., Chicago, IL, USA) was used to generate figures and to run an initial analysis. Independent samples *t*-test was performed to determine the significance *p* < 0.05 of the mean CFU between the sublocations and the seasons of each center. Metagenomic analysis was performed using the CLC Workbench V.20.0.4 (CLC, Qiagen). Operational taxonomic unit (OUT) clustering was performed to determine the genera in each sample and their abundance. Shannon alpha diversity was computed per pooled samples, and a Kruskal–Wallis test was performed to compare the alpha diversities between the hot and cold seasons. The beta diversity was computed using Bray–Curtis dissimilarity and principal coordinate analysis (PCoA). A PERMANOVA test was performed to determine the statistical value of the variation. A probability value (*p*-value) less than 0.05 was considered statistically significant.

### 2.3. Methicillin-Resistant Staphylococcus Aureus (MRSA) Detection

The Andersen impactor was used, as described above, to detect the presence of MRSA in the air. However, instead of nutrient agar, the impactor was loaded with chromatic MRSA agar (Liofilchem^®^, Italy); all plates were immediately transferred into the lab and incubated at 37°C for three days. Presumptive MRSA colonies were identified by color according to each manufacturer’s instructions: Liofilchem^®^ Chromatic MRSA, positive results give mauve colonies. For further identification, colonies were isolated and tested by a latex test (Staph Latex Kit, Liofilchem, Italy) and by Gram staining.

### 2.4. Non-Cultivable Airborne Bacteria

An SKC button sampler was used to collect non-cultivable bacteria with an airflow of 4 L/min (SKC Inc., Eighty-Four, UK). The button sampler was equipped with 25 mm-diameter cellulose ester filters (1.2 µm pore diameter) and the air was collected for 8 h (total air volume of approximately 1.92 m^3^) [24]. The cellulose filters were immediately frozen at −20 °C after sampling.

#### DNA Extraction

The cellulose filters were vortexed in PBS for 5 min. The ZymoBIOMICS™ kit was used for DNA extraction of bacteria according to the manufacturer’s instructions with the following modifications: air filters were incubated with a PBS solution in a 65 °C water bath for 15 min, then centrifuged for 20 min at maximum speed to maximize the amount of precipitated DNA. The DNA was quantified using NanoDrop (ThermoScientific, Wilmington, DE, USA). However, the quantities were extremely low, thus we could not proceed with the 16S rRNA gene sequencing from the filters.

## 3. Results

### 3.1. Airborne Bacterial Concentration, CFU/m^3^

For cultivable airborne bacteria, 112 air samples were collected from the lobby and the triage room indoor area, and 80 air samples from the outdoor area were collected from the four PHCCs. Table 2 is the detailed total bacterial count recorded during the collection period. The shaded numbers are the data points that exceeded the recommended levels of airborne bacteria by WHO (300 CFU/m^3^ of air). The highest total bacterial count was recorded in the triage room of AR-HC in September 2020 (1097.8 CFU/m^3^). In contrast, the lowest bacterial load was found in the triage room of QU-HC in January 2021 (45.9 CFU/m^3^). It is worth mentioning that all data from the AR-HC lobby were above 300 CFU/m^3^.

**Figure 3:**
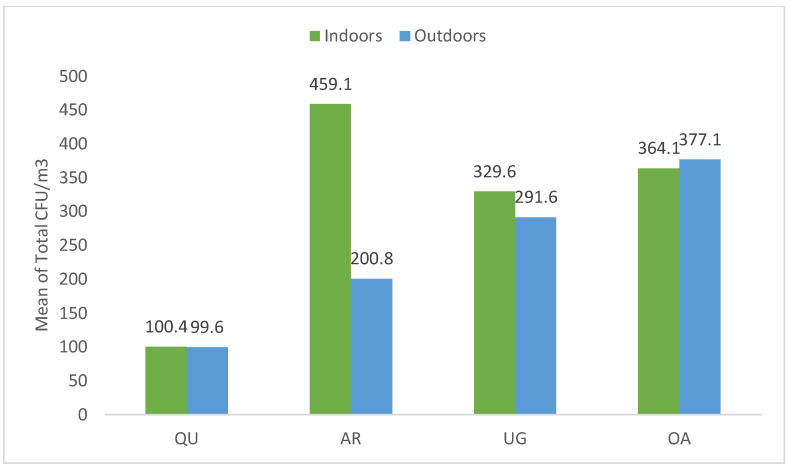
The mean of total indoor and outdoor airborne bacterial concentration (CFU/m^3^) at all health centers.

The total CFU/m^3^ mean of the indoor and outdoor areas of all centers ranged between 99–459 CFU/m^3^, and the total mean for each health center was as follows: QU-HC (100.4 and 99.6 CFU/m^3^), AR-HC (459–200 CFU/m^3^), OA-HC (364–377 CFU/m^3^), and UG-HC (329–291 CFU/m^3^), respectively. Notably, QU-HC had the lowest airborne bacteria concentration in both the indoor and outdoor areas with 100.4 and 99.6 CFU/m^3^, respectively. In comparison, the AR-HC indoor areas had the highest mean of bacterial concentration, with 459.1 CFU/m^3^. In addition, the OA-HC outdoor area recorded the highest mean of bacterial concentration, with 377.1 CFU/m^3^ (Figure 3).

The mean of total CFU/m^3^ of indoor and outdoor areas was used to calculate the indoor to outdoor bioaerosol concentration ratio (I/O). This ratio explains the relationship between indoor and outdoor particle concentrations and it estimates the emissions sources of bioaerosol. The I/O ratio is defined as: [25,26].

$$I/O\;\mathrm{ratio}=\frac{C.\mathrm{in}}{C.\mathrm{out}}$$

where *C.*_in_ and *C.*_out_ are the indoor and outdoor airborne bacteria (CFU/m^3^) concentrations, correspondingly. The I/O ratio of QU-HC, AR-HC, and UG-HC was ≥1 and was 1.0, 2.2, and 1.1, respectively, except OA-HC that had a 0.96I/O ratio which is <1.

**Figure 4:**
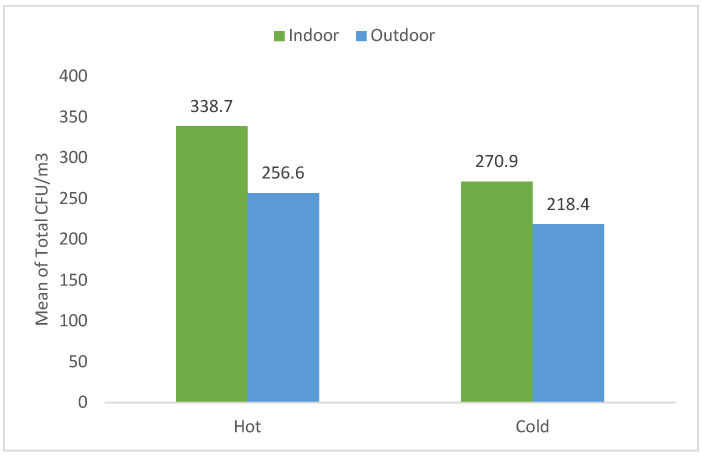
The total mean of indoor and outdoor airborne concentration (CFU/m^3^) during the hot and cold seasons at all health centers.

Figure 4 shows the seasonal variation of total CFU/m^3^ mean of indoor and outdoor areas of all health centers. The maximum bacterial concentration was recorded in indoor hot and cold seasons, where the total colony count was 338.7–270.9 CFU/m^3^, respectively, while in the outdoor area, the total mean bacterial concentration was 256.6–218.4 CFU/m^3^, respectively.

**Figure 5:**
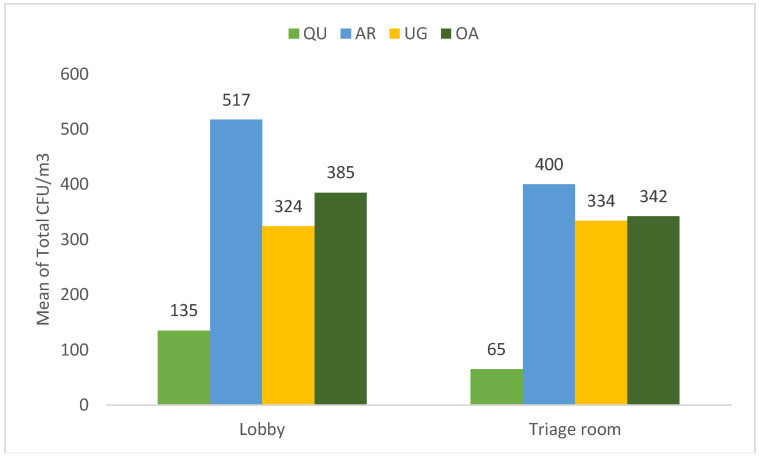
The total mean airborne concentration of indoor areas (lobby and triage room) at all health centers.

To thoroughly explore the indoor environment of all PHCCs, the total mean of bacterial count CFU in the lobby and the triage room was investigated (Figure 5). As a result, the lobby and the triage room of QU-HC recorded a significantly low bacterial concentration *p* < 0.05, with a total mean of (135–65 CFU/m^3^), respectively. In addition, the highest bacterial count (517–400 CFU/m^3^) was recorded in both areas at AR-HC, respectively. However, there was no significant difference between the lobby and the triage room of the three investigated health centers.

**Figure 6:**
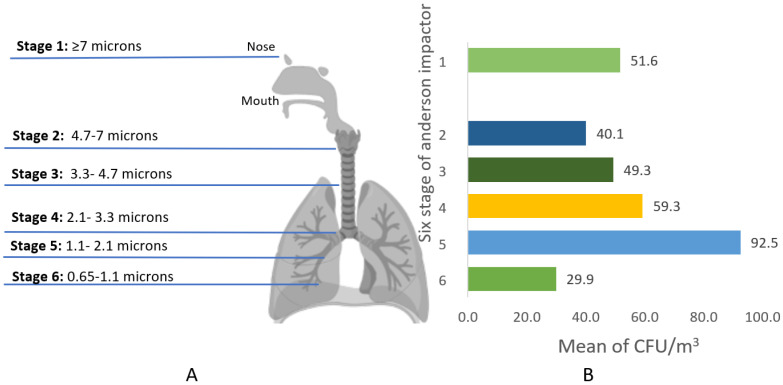
(**A**) Simulation of the size distribution of airborne bacteria deposition in the human respiratory system in relation to the Andersen impactor stages. (**B**) The mean of indoor total air bacterial concentrations (CFU/m^3^) per stage of the Anderson impactor across all samples.

To investigate the size distribution level of the airborne bacteria according to the human respiratory system, the prevalent size range of the collected samples from all studied PHCCs was between 1.1–2.1 µm in diameter with an average colony count of 92.5 CFU/m^3^, representing stage 5 of the Anderson six-stage impactor (Figure 6).

As for the non-cultivable bacteria, 85 air filters were collected from the indoor areas and 42 air filters from the outdoor areas. However, due to the extremely low DNA concentration extracted from the filter, these samples were excluded from the study [27,28].

### 3.2. Bacterial Composition of the Samples

**Figure 7:**
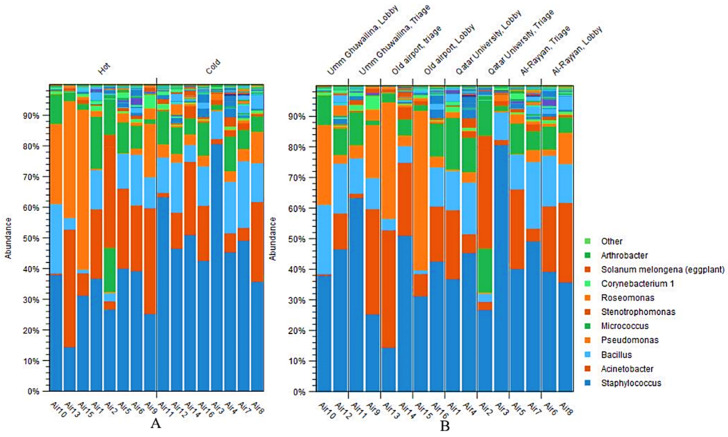
The 10 most relative abundant bacterial genera classified by season (**A**) and location (**B**).

Overall, ten bacterial genera were identified in the samples, including Staphylococcus, Acinetobacter, Bacillus, Pseudomonas, Micrococcus, Stenotrophomonas, Roseomonas, Corynebacterium1, Solanum Melongena, and Arthrobacter. The Staphylococcus genus was the most abundant in most cases, ranging from 15–80%, followed by Acinetobacter, Pseudomonas, and Bacillus (Figure 7). Interestingly, the relative abundance of Staphylococcus was generally higher in the cold season compared with the hot season, while the abundances of Acinetobacter and Pseudomonas show the opposite trend. Nevertheless, there is variability in the relative abundances between locations and seasons within the same site. Notably, Pseudomonas was the second highest distributed bacterial genus in OA-HC during the hot season after Staphylococcus.

**Figure 8:**
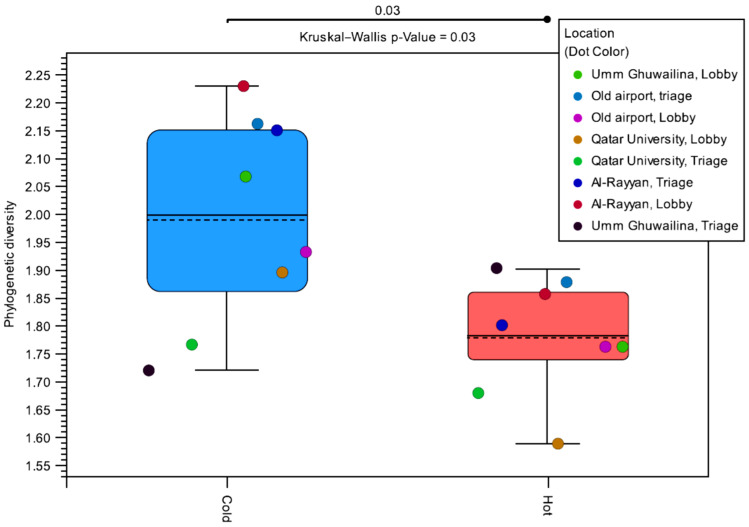
Distribution of the alpha diversities by season. Alpha diversity represents the diversity within each sample through the number of bacterial species in each sample weighted by their abundance. Each pair of points sharing the same color was obtained in different seasons from the same location.

The alpha diversity was measured per sublocation and per season to determine the variation in genus diversity over seasons. The distribution of alpha diversities is shown in Figure 8. The alpha diversity was higher in the cold season for each location, except for the UG-HC triage room. Overall, the median alpha diversity was higher in the cold season (*p*-value = 0.03). Additionally, there was more variability in the alpha diversity of samples obtained in the cold season compared with the hot season. Stratifying by location instead of the season shows no significant difference, indicating that the effect is seasonal rather than location dependent.

**Figure 9:**
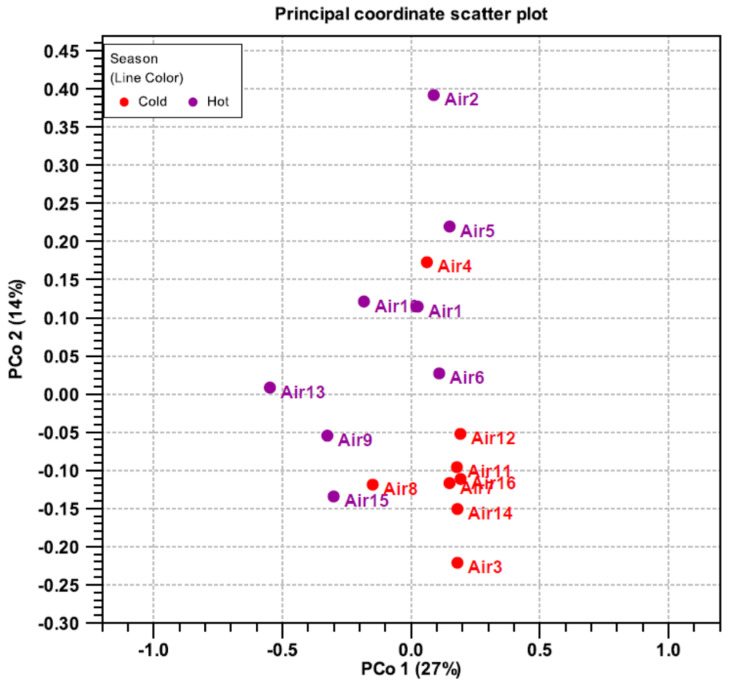
Diversity principal coordinate analysis of cultivable airborne bacteria collected from four PHCCs and analyzed based on 16SrRNAA sequencing during the hot and cold seasons.

Beta diversity was computed using a principal coordinate analysis (PCoA) to assess the diversity between samples in the hot and cold season. Similar samples cluster together and share common characteristics (Figure 9). Most samples from the cold season form a single set. In contrast, the representatives from the hot season are spread further apart. Markedly, Air 4 and Air 8 (both cold season samples) are more like the hot season samples than other cold season samples. These samples were collected from the lobby of QU-HC and AR-HC, respectively.

**Figure 10:**
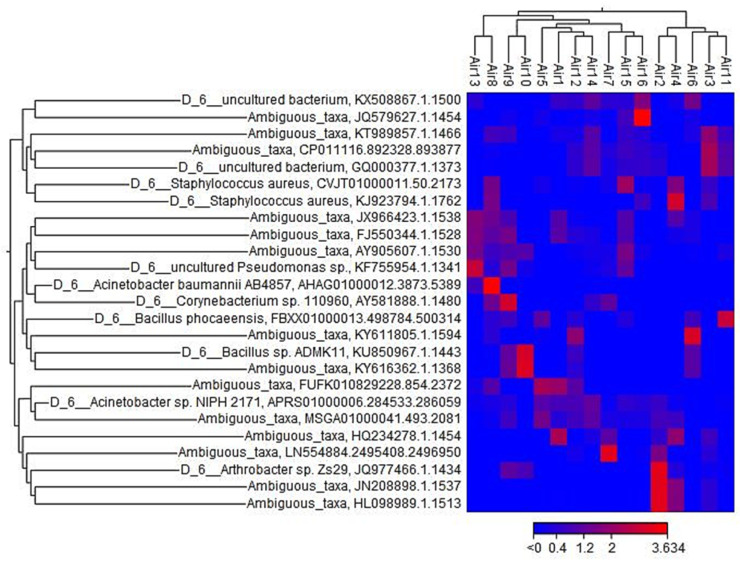
Heatmap of relative abundance of the bacterial sample sorted by location (top cluster). Hierarchical clustering patterns of OTUs of bacteria (left cluster).

The heatmap (Figure 10) reveals the relative abundance of the bacterial sample sorted by location. Remarkably, the hierarchical clustering at the top represents two similar clusters. The first cluster was a combination of samples Air3, Air2, and Air4 collected from QU-HC and samples Air6 and Air11 from AR-HC and UG-HC, respectively, during the hot and cold seasons. A second cluster represents a similar combination of the rest of the samples. Another hierarchical clustering on the left side of the heatmap shows the operational taxonomic unit (OTUs) abundances at various taxonomical levels. Relative to the OTUs level, ambiguous taxa were the most taxa presented.

## 4. Discussion

Maintaining excellent indoor air quality (IAQ) in the hospital environment and eliminating potential airborne bacterial contamination are essential points to protect respiratory health and to prevent the spread of disease. Therefore, in this study, we assessed the concentrations of airborne bacteria in various locations within four PHCCs in the state of Qatar. Additionally, we connected this to the seasonal variations and gauged the most prevalent bacterial genera found in the health care environments.

It is worth mentioning that 46.4% of the total indoor airborne bacterial concentrations were above the WHO-recommended level of 300 CFU/m^3^, which advised that the total microbial loads in an indoor environment should not exceed 300 CFU/m^3^ and 100 CFU/m^3^ in an indoor environment occupied by immunocompromised patients [17]. Similarly, multiple studies have been done in the Middle East region assessing the levels of airborne bacteria of health care settings and have observed high concentrations of CFU in hospital indoor environments [29,30,31,32,33]. Mirhoseini studied four hospitals in Iran: the CFU levels ranged between 99 and 1079 CFU/m^3^ in multiple sublocations of the hospitals [32]. Similarly, another study in Gaza City found significant CFU levels, with an average of 780 CFU/m^3^ [30].

The high levels of airborne bacteria in most of the investigated health centers are mainly caused by anthropogenic activities in indoor areas [34]. The number of occupants, poor ventilation systems, and the size and buildings’ age are possible factors contributing to the levels of bacterial contamination in indoor air [12,35]. Other reasons that could influence the IAQ are the structure of the building and the habits of the residents [36]. Thus, levels of indoor contamination differ from one location to another according to the type and number of activities carried out in each place.

Moreover, indoor to outdoor I/O ratios in OA-HC were <1.0, meaning outdoor air was the main source of bioaerosols inside the health center. Hence, this contamination could be attributed to natural ventilation, organic materials such as flowers, and food derived from the outdoor environment by visitors [18,37]. In addition, QU-HC, UG-HC, and AR-HC I/O ratios were greater than 1.0, indicating that the bacterial load in the interior area was obtained from the indoor environment itself, such as the crowdedness of people and their indoor activities. This is consistent with the findings in a hospital in Iran, where they recorded that the CFU mean value of the indoor area was higher than the outdoor area, 49.1 ± 23.8 and 47.1 ± 21.5 CFU/m^3^, respectively [32].

Particle deposition size is a crucial parameter influencing the result of human exposure to airborne microorganisms. The most dominant size range of airborne bacteria found in the health centers was 1.1–2.1 µm in diameter. Similarly, Onklay et al., demonstrate that 75% of the total bacteria in the studied hospital in Thailand with a diameter size of 1.1–2.1 µm are capable of being deposited in the lower respiratory tract, including the alveoli and the bronchi [37]. Most likely, this size of bacteria is contaminating the indoor air of health care facilities and patients are exhaling these bacteria through coughing and sneezing [38], thus posing health risks of acquiring nosocomial infections among patients [39,40].

Moreover, in this study, the most frequently identified airborne bacteria genera were *Staphylococcus*, *Acinetobacter*, *Bacillus*, and *Pseudomonas*. It is not surprising that *Staphylococcus* was one of the most dominant genera of the bacteria genera as it is also identified in high concentration in hospital air in several other studies [40,41,42]. A study conducted in an Iran education hospital found that 95% of the isolated bacteria belonged to the genera *Staphylococcus* [32]. Qudiesat et al., also conducted a study in an airborne contamination in hospital and reported that *Staphylococcus aureus* was the dominant bacteria isolated from a governmental hospital in Zarqa, Jordan [42]. *Staphylococcus* is well known to be transmitted into the air from human skin, oral and nasal surfaces, and hair and is widely spread in indoor hospital environments [39,40]. Furthermore, a study in an Ethiopian university hospital revealed a high prevalence of 42% of the genus *Acinetobacter*, justifying this persistence to its great survival ability in the indoor environment [43]. This can potentially cause hospital infections transmitted via the air [44,45]. Another relevant microorganism is *Bacillus*, considered the most common bacteria in intensive care units during the previous two decades and recognized as a nosocomial pathogen associated with several outbreaks in immunocompromised patients [46]. Similar to our findings, *Pseudomonas* was reported in the air of a hospital ward in a Tehran teaching hospital, in the gynecology room, the main operating room, and on the instrument trolley [47]. As well, *Pseudomonas* is considered one of the most significant genera of nosocomial pathogens and was the most prevalent bacteria in all operating theatres in the Matinyi et al., study [48] and was identified in other studies [49,50].

Furthermore, fluctuations in bacterial diversity in comparison to seasonal variation were detected. A high variation of airborne bacteria was recorded in the cold season rather than the hot season. Likewise, many studies reported that winter was the season with the highest airborne bacteria [51,52]. This surge may be attributed to various factors, including that in the winter, residents spend more time indoors, windows are closed, and ventilation is inadequate; furthermore, the temperature and relative humidity are more supportive to bacterial development indoors than outside [53]. Additionally, we investigated the relationship between the bacterial concentration and the seasonal variation of the studied health centers. Consequently, there were no significant differences between the hot and cold seasons in the indoor and outdoor areas, unlike two studies conducted in hospitals in Korea and Portugal that revealed that the highest bacterial concentration was recorded during the summer [18,54].

## 5. Limitations

The main limitation was that we could not extract sufficient DNA from the SKC air filters used to obtain the non-cultivable bacteria, which might have been a valuable addition for comparing the cultivable and non-cultivable bacteria perspectives; some bacteria may have been missed because not all bacteria thrive in agar media.

## 6. Conclusions

This is the first study in Qatar to assess airborne bacterial concentrations in Primary Health Care Centers and, thus, provides vital baseline and preliminary information in this regard. The results of this study emphasize the high level of total bacterial concentration in the indoor environment. In addition, the data highlight that *Staphylococcus*, *Acinetobacter, Bacillus*, and *Pseudomonas* were the most common opportunistic pathogens identified within the health care facilities. These bacteria can threaten immunocompromised patients’ health and may contribute to the spread of infection. The number of CFU in most health centers exceeded the WHO recommendation level of indoor bacterial contamination. Therefore, an IAQ guideline should be implemented and an assessment of airborne bacteria concentration in indoor environments should be frequently carried out, especially in the hospital environment. These regulations can provide a baseline for air contamination measurements and a better understanding of seasonal variations, thus allowing hospitals to avoid/ be ready for the infection spreading peaks.

## Figures and Tables

**Table 1:** The 16 pooled cultivable airborne bacteria collected from indoor sublocations in the mornings at the four PHCC centers and used for 16S rRNA metagenomic sequencing.

Sample Number	Sample Location	Season *
Air 1	Qatar University HC, Lobby	Hot
Air 2	Qatar University HC, Triage	Hot
Air 3	Qatar University HC, Triage	Cold
Air 4	Qatar University HC, Lobby	Cold
Air 5	Al-Rayyan HC, Triage	Hot
Air 6	Al-Rayyan HC, Lobby	Hot
Air 7	Al-Rayyan HC, Triage	Cold
Air 8	Al-Rayyan HC, Lobby	Cold
Air 9	Umm-Ghuwailina HC, Triage	Hot
Air 10	Umm-Ghuwailina HC, Lobby	Hot
Air 11	Umm-Ghuwailina HC, Triage	Cold
Air 12	Umm-Ghuwailina HC, Lobby	Cold
Air 13	Old-airport HC, Triage	Hot
Air 14	Old-airport HC, Triage	Cold
Air 15	Old-airport HC, Lobby	Hot
Air 16	Old-airport HC, Lobby	Cold

* The hot season corresponds to the months from March to September and the cold season resembles the months between October and February

**Table 2:** Total CFU count of airborne bacteria during the period of August 2020–March 2021 for the four health centers.

	QU-HC			AR-HC			UG-HC			OA-HC		
	Triage	Lobby	Outdoor	Triage	Lobby	Outdoor	Triage	Lobby	Outdoor	Triage	Lobby	Outdoor
August 2020 trial 1	52.9	129.0	67.3	260.3	361.6	133.1	426.4	239.1	179.0	274.4	458.2	240.3
August 2020 trial 2	53.0	128.4	68.3	247.3	352.2	109.5	239.1	426.4	179.0	274.4	458.2	240.3
September 2020 trial 1	64.8	96.6	36.5	281.5	387.5	113.1	22.4	15.3	3.5	25.9	63.6	22.4
September 2020 trial 2	64.8	172.0	106.0	1097.8	922.3	349.8	353.4	372.2	199.1	345.1	779.7	537.1
October 2020 trial 1	63.6	166.1	66.0	325.1	405.2	67.1	204.9	286.2	320.4	407.5	459.4	241.5
October 2020 trial 2	94.2	129.6	84.8	580.7	747.9	131.9	541.8	335.7	394.6	341.6	342.8	368.7
November 2020 trial 1	50.6	164.9	216.7	339.2	773.9	289.8	384.0	577.1	387.5	468.8	699.6	475.9
November 2020 trial 2	60.1	181.4	248.5	875.1	631.3	253.2	455.8	345.1	260.3	401.6	308.6	448.8
December 2020 trial 1	49.5	168.4	66.0	424.0	318.0	98.9	540.6	285.0	305.1	169.6	360.4	504.1
December 2020 trial 2	46.0	103.7	96.6	445.2	592.5	248.5	255.6	359.2	151.9	378.1	263.8	308.6
January 2021 trial 1	45.9	110.7	95.4	431.1	621.9	255.6	186.1	168.4	129.6	364.0	289.8	193.2
January 2021 trial 2	56.5	164.9	94.2	428.7	505.3	170.8	221.4	278.0	116.6	486.5	465.3	293.3
February 2021 trial 1	57.7	111.9	111.9	175.5	408.7	234.4	544.2	580.7	308.6	484.1	319.2	513.5
March 2021 trial 1	116.6	137.8	61.2	160.2	422.9	261.5	217.9	170.8	439.3	289.8	288.6	566.5

## Data Availability

Not applicable.
